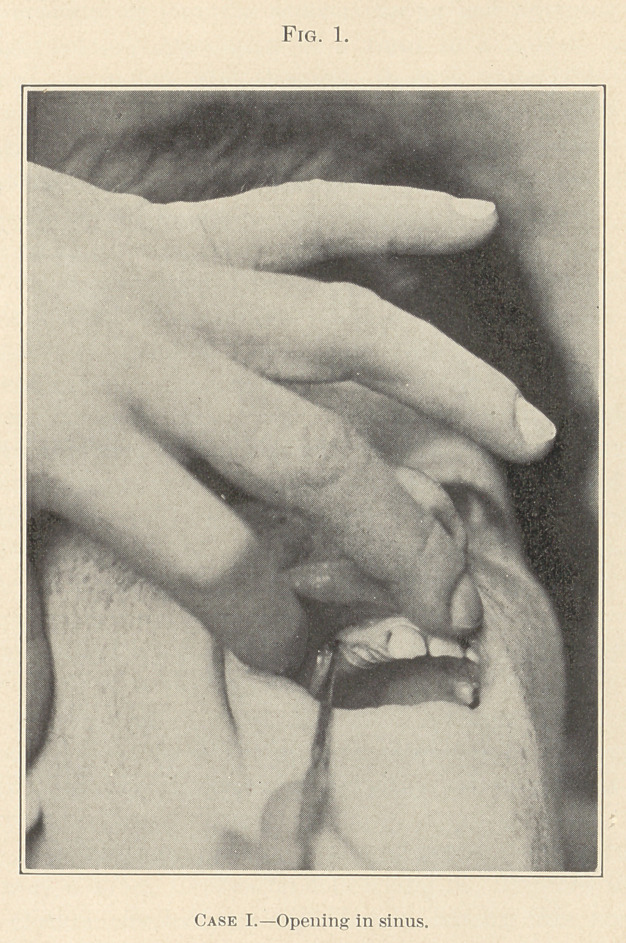# Cases of Maxillary Sinus Derangement

**Published:** 1903-12

**Authors:** Percy R. Howe

**Affiliations:** Boston, Mass.


### International Dental Journal.

VOL. XXIV. DECEMBER, 1903. No. 12.

### Original Communications.[1]

### CASES OF MAXILLARY SINUS DERANGEMENT.

BY PERCY R. HOWE, M.A., D.D.S., BOSTON, MASS.

OF the accessory sinuses of the nose, the maxillary sinus is by far most frequently the seat of inflammatory affections. This is in part due to the relation of the teeth to this cavity, and therefore has always been regarded as a subject of great interest and importance to the dental profession. Within the last few years the research of eminent specialists has added much to our general knowledge of this branch of science, and at the same time shown many other sources of infection, as well as the anatomical construction which permits of such infection. The works of Dr. A. Logan Turner, of Dr. Cryer, Lindenthal, Weichselbaum, and E. Frankel are among the most important on this subject, and present facts with which the dental practitioner should be familiar.

In attacks of coryza, influenza, pneumonia, scarlet fever, and diphtheria the mischief to be expected is that the sinuses will be infected by the specific bacteria of these diseases; for these have been discovered in these cavities, both in living subjects and in

[1] The editor and publishers are not responsible for the views of authors of papers published in this department, nor for any claim to novelty, or otherwise, that may be made by them. No papers will be received for this department that have appeared in any other journal published in the country.

cadavera. Lindenthal finds the influenza bacillus present in the sinuses of many subjects that he has examined, and he believes that this is, of itself, sufficient to produce pus; so that it may be accepted as a settled thing that inflammation and suppuration of the sinuses are much more common than was formerly supposed, and present themselves either in an acute or a chronic form.

In many cases the ostium of the affected part remains patent, the secretions drain off, and a spontaneous cure takes place; but severe trouble may arise if the ostium becomes closed by reason of the swelling of its mucous membrane. Post-mortem examinations have shown that from twenty-five to forty-five per cent. of unselected cases have pus in the sinuses. Although this may be in part a result of the fatal diseases, yet investigators feel warranted in saying that a large per cent. of diseased sinuses remain without treatment because unrecognized.

Dr. A. Logan Turner tells us that pus may be secreted for months, and even years, in one of the accessory sinuses with almost entire absence of pain or swelling to suggest the existence of such an unhealthful and inflammatory condition; and hence we get the term "latent empyema." The symptoms of chronic suppuration in the sinuses are nasal discharge, cough, dryness of the throat, huskiness of the voice. Indigestion may also result from an enforced swallowing of the offensive secretion.

Impaired vision is not as often met with in maxillary sinus suppuration as it is in disease of the ethmoidal and sphenoidal cavities; however, orbital complications may also occasionally accompany maxillary sinus disease and the cause not be suspected. One of the cases that I have treated was of this nature, and I have ventured to give an account of it with some others.

Case I.—The patient, a young lady seventeen years of age, came to me for dental work. On examination of her mouth, I found the teeth missing in the right superior maxilla, with the exception of the right central incisor and the point of a tooth protruding high up and back in the canine fossa. The latter was movable, and with probe and fingers was easily taken out, disclosing a free opening into the maxillary sinus. The tooth itself was an undeveloped cuspid with the root absorbed, having the appearance of an exfoliated deciduous tooth. The sinus was high-vaulted and large, especially for the age of the patient. Its walls were intact, and gave to the probe no evidence of a carious or necrotic condition. The

FIG. 1.

CASE I.—Opening in sinus.

thin nasal wall, which, it will be remembered, has in certain instances been declared to be membranous, seemed hard and firm, as did also the orbital plate.

By means of an ear speculum and reflected light it was possible to examine visually this cavity sufficiently to see that, while there was no evidence of pus, the mucous lining had anything but the thin, light-colored appearance of a sinus in a normal condition. Thickened, congested, and sluggish, it gave undeniable evidence of having at one time been the seat of intense infection and inflammation. The ostium maxillare was also of a large size, as evidenced by the ease with which fluids passed from the sinus into the nose during syringing.

The right eye was nearly sightless, the patient being scarcely able to distinguish light from darkness. Five years previous to this time she had suffered excruciating pain in this region, and had been under treatment of a physician, who had referred her to a prominent eye specialist, but the cause of the trouble escaped his detection. The sight gradually failed and the pain subsided.

When this side of the patient's face was causing her intense pain, she had had teeth extracted in hope of getting relief. These extractions cleared all her superior teeth on the afflicted side except those already mentioned. With the exception of a few simple cavities, her teeth on the left superior maxilla were intact, and her inferior teeth as well.

Treatment of the sinus by enlarging the opening, curetting the walls, packing with sterile gauze, followed later by gentle flushing with a boracic acid solution, has much improved the condition. The opening has closed and the eye recovered to such an extent that, with the aid of proper glasses, the patient is able to read ordinary print.

The appearance of the eye is almost normal, although the cut may convey a wrong impression concerning it. To show the nicety of the closure effected was my idea in having this photograph taken. (See Fig. 1.)

CASE II.—The patient is a young lady twenty years of age. When seen by me she had a temperature of 103°, rapid pulse, and was suffering intense pain in the left side of her face. By her physician's advice her teeth had been twice examined by her dentist, and had been pronounced sound. When I examined her mouth, a slight soreness of her superior left wisdom-tooth was apparent, and the nasal wall was also sore and swollen. No discharge into the meatuses of the nose had been noticed.

Although the symptoms were not as decisive as they might have been, the severity of the suffering and the general febrile condition of the patient were sufficient proof to me that the sinus was affected and that an opening into it was indicated. Inasmuch as the wisdom-tooth was sore to percussion, I concluded that this was the nearest approach to the seat of the trouble, and accordingly extracted it and through its socket entered the sinus. This was found to be intensely inflamed. Douching with the boracic acid solution reduced this inflammation, caused the pain to cease, and in a few days ended the whole trouble.

This case seemed to have some peculiar characteristics. It seemed to be infected from causes other than the teeth; and yet there was no catarrhal state, no coryza, no malaise condition of the patient to which this trouble could be attributed. The teeth were perfect, the mouth had had the best of care, and was as cleanly and hygienic as could be desired. It is of interest to note that the patient's mother had been operated upon twice for pus in the mastoid cells.

Case III. — Mrs. D., aged forty-five years, had long been troubled by a fetid discharge from the nose, and had been under continued treatment for nasal catarrh. The teeth were sound. Examination of the nose showed pus in the middle meatus; and after cleansing and trying the posture test for a few minutes, the presence of more pus was observed on the affected side. Opacity in the region of the antrum, in response to illumination of the face, convinced us of the location of the trouble. An opening through the canine fossa was effected, the teeth being *in situ,* through which a considerable quantity of offensive pus was evacuated, as we had anticipated. Treating by lavage for a considerable period, a cure was gradually brought about; at least, after two years, the patient has complained of no further trouble, and her catarrh has so subsided that she is no longer conscious of it.

Case IV.—This was the case of a man who had been previously but unsuccessfully operated upon,—a man fifty-five years of age, who, having lost his teeth, was wearing an artificial plate. A large opening had been made into the antrum and a drainage-tube inserted. So far as we might judge from appearances, a permanent state of infection existed. For eight years this gentleman had been douching and syringing this cavity and taking most particular pains to keep this part in an antiseptic condition.

This case was not mine to prescribe for, but the patient wished me to examine his mouth. The treatment employed and the resulting condition of the patient's jaw seemed worth describing, inasmuch as this seemed only a fair representative of too many cases of this kind which we have seen, cases which have led us to be no advocate of large openings, irritating washes, rough curettages, or the insertion of mechanical devices for drainage. Such methods defeat the very ends which they aim to attain. Here was a need, a treatment, but no cure.

On operative procedure in such cases much has been written, but individual conditions must always govern our choice of methods, and not hard-and-fast theories. If an opening through the canine fossa is deemed best, let there be a clean incision from the canine eminence to the molar process of the superior maxillary bone, a lifting back of the periosteum, and an opening drilled a trifle larger than a lead-pencil. For curettage, if necessary, the use of a flexible, ringed, nasal curette is very convenient. An opening through a tooth-socket may need to be a little larger on account of the thickness of the tissue from the gingival margin of the alveolus to the floor of the sinus, but it should always be made with the idea of eventually effecting a closure of the parts.

Bleeding is controlled with throat and cheek sponges, with packings, and with adrenalin chloride. The after-treatment can be with aseptic gauze, changed when necessary, or by the more common lavage treatment.

## Figures and Tables

**Fig. 1. f1:**